# *N*′-[(2-Hy­droxy­naphthalen-1-yl)methyl­idene]-4-nitro­benzohydrazide

**DOI:** 10.1107/S1600536811045685

**Published:** 2011-11-12

**Authors:** Yan An, Xiaofeng Li, Yingjie Zhang

**Affiliations:** a Institute of Marine Materials Science and Engineering, Shanghai Maritime University, Shanghai 201306, People’s Republic of China

## Abstract

In the title mol­ecule, C_18_H_13_N_3_O_4_, the hy­droxy group is involved in the formation of an intra­molecular O—H⋯N hydrogen bond. The dihedral angle between the planes of the benzene ring and the naphthyl ring system is 9.0 (2)°. In the crystal, mol­ecules are linked through N—H⋯O hydrogen bonds into chains along the *c* axis.

## Related literature

For recently published crystal structures of hydrazone compounds, see: Horkaew *et al.* (2011 ▶); Fun *et al.* (2011 ▶); Su *et al.* (2011 ▶); Zhi *et al.* (2011 ▶).
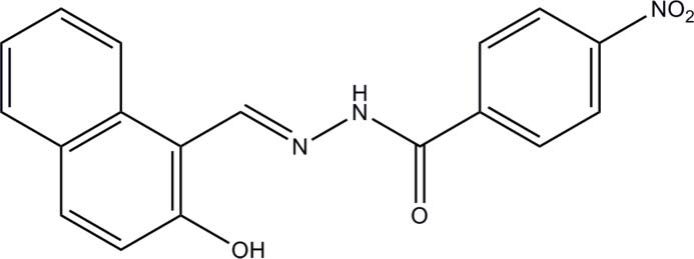


## Experimental

### 

#### Crystal data

C_18_H_13_N_3_O_4_
*M* *_r_* = 335.31
Monoclinic, 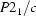

*a* = 11.208 (3) Å
*b* = 15.432 (3) Å
*c* = 8.982 (2) Å
β = 90.701 (2)°
*V* = 1553.4 (6) Å^3^
*Z* = 4
Mo *K*α radiation
μ = 0.10 mm^−1^
*T* = 298 K
0.20 × 0.20 × 0.17 mm

#### Data collection

Bruker SMART 1K CCD area-detector diffractometer
Absorption correction: multi-scan (*SADABS*; Sheldrick, 1996 ▶) *T*_min_ = 0.980, *T*_max_ = 0.983
8323 measured reflections
2817 independent reflections
1564 reflections with *I* > 2σ(*I*)
*R*_int_ = 0.059

#### Refinement

*R*[*F* ^2^ > 2σ(*F* ^2^)] = 0.069
*wR*(*F* ^2^) = 0.127
*S* = 1.02
2817 reflections
232 parameters
2 restraints
H atoms treated by a mixture of independent and constrained refinement
Δρ_max_ = 0.18 e Å^−3^
Δρ_min_ = −0.24 e Å^−3^

### 

Data collection: *SMART* (Bruker, 2007 ▶); cell refinement: *SAINT* (Bruker, 2007 ▶); data reduction: *SAINT*; program(s) used to solve structure: *SHELXS97* (Sheldrick, 2008 ▶); program(s) used to refine structure: *SHELXL97* (Sheldrick, 2008 ▶); molecular graphics: *SHELXTL* (Sheldrick, 2008 ▶); software used to prepare material for publication: *SHELXTL*.

## Supplementary Material

Crystal structure: contains datablock(s) I, global. DOI: 10.1107/S1600536811045685/cv5182sup1.cif

Structure factors: contains datablock(s) I. DOI: 10.1107/S1600536811045685/cv5182Isup2.hkl

Supplementary material file. DOI: 10.1107/S1600536811045685/cv5182Isup3.cml

Additional supplementary materials:  crystallographic information; 3D view; checkCIF report

## Figures and Tables

**Table 1:** Hydrogen-bond geometry (Å, °)

*D*—H⋯*A*	*D*—H	H⋯*A*	*D*⋯*A*	*D*—H⋯*A*
O1—H1⋯N1	0.86 (1)	1.85 (2)	2.599 (3)	144 (3)
N2—H2⋯O2^i^	0.90 (1)	2.06 (1)	2.923 (3)	160 (3)

Symmetry code: (i) 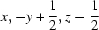.

**Fig. 1.**
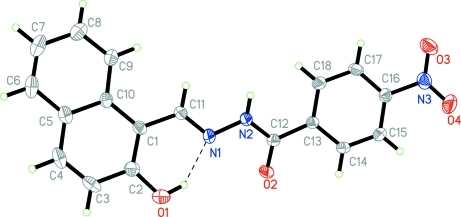
The molecular structure of (I) with the displacement ellipsoids drawn at the 30% probability level. Intramolecular O—H···N hydrogen bond is shown as a dashed line.

**Fig. 2.**
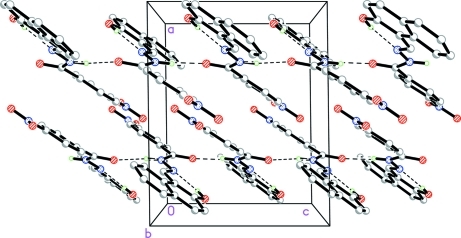
A portion of the crystal packing viewed approximately along the b axis. Intermolecular N—H···O hydrogen bonds are shown as dashed lines. H-atoms not involved in the hydrogen bonding have been omitted.

## supplementary crystallographic information

### Comment

As a continuation of structural studies of hydrazone compounds (Horkaew *et al.*, 2011; Fun *et al.*, 2011; Su *et al.*, 2011; Zhi *et al.*, 2011), we present here the title new hydrazone compound (I).

In (I) (Fig. 1), intramolecular O—H···N hydrogen bond (Table 1) and conjugation effects of the molecule lead to the flattening of the whole molecule. The dihedral angle between the benzene ring and the naphthyl ring is 9.0 (2)°. The bond lengths and angles are normal and comparable to those observed in the related structures  (Horkaew *et al.*, 2011; Fun *et al.*, 2011; Su *et al.*, 2011; Zhi *et al.*, 2011).

In the crystal structure of the compound, molecules are linked through intermolecular N—H···O hydrogen bonds (Table 1) to form chains along the *c* axis (Fig. 2).

### Experimental

Equimolar quantities (0.5 mmol each) of 2-hydroxy-1-naphthaldehyde and 4-nitrobenzohydrazide were mixed in 30 ml me thanol. The mixture was stirred at reflux for 30 min and cooled to room temperature. Yellow block-shaped single crytals were formed by slow evaporation of the solvent in air.

### Refinement

The N- and O-bound H atoms were located in a difference Fourier map and were refined with distance restraints [N—H = 0.90 (1) Å, O—H = 0.85 (1) Å], and with *U*_iso_(H) fixed to 0.08. The remaining H atoms were positioned geometrically and refined using a riding model, with C—H = 0.93 Å, and with *U*_iso_(H) = 1.2*U*_eq_(C).

### Crystal data

**Table:**

C_18_H_13_N_3_O_4_	*F*(000) = 696
*M**_r_* = 335.31	*D*_x_ = 1.434 Mg m^−^^3^
Monoclinic, *P*2_1_/*c*	Mo *K*α radiation, λ = 0.71073 Å
*a* = 11.208 (3) Å	Cell parameters from 1804 reflections
*b* = 15.432 (3) Å	θ = 2.2–28.2°
*c* = 8.982 (2) Å	µ = 0.10 mm^−^^1^
β = 90.701 (2)°	*T* = 298 K
*V* = 1553.4 (6) Å^3^	Block, yellow
*Z* = 4	0.20 × 0.20 × 0.17 mm

### Data collection

**Table:**

Bruker SMART 1K CCD area-detector diffractometer	2817 independent reflections
Radiation source: fine-focus sealed tube	1564 reflections with *I* > 2σ(*I*)
graphite	*R*_int_ = 0.059
ω scan	θ_max_ = 25.5°, θ_min_ = 2.3°
Absorption correction: multi-scan (*SADABS*; Sheldrick, 1996)	*h* = −13→13
*T*_min_ = 0.980, *T*_max_ = 0.983	*k* = −18→16
8323 measured reflections	*l* = −10→10

### Refinement

**Table:**

Refinement on *F*^2^	Primary atom site location: structure-invariant direct methods
Least-squares matrix: full	Secondary atom site location: difference Fourier map
*R*[*F*^2^ > 2σ(*F*^2^)] = 0.069	Hydrogen site location: inferred from neighbouring sites
*wR*(*F*^2^) = 0.127	H atoms treated by a mixture of independent and constrained refinement
*S* = 1.02	*w* = 1/[σ^2^(*F*_o_^2^) + (0.0411*P*)^2^ + 0.3228*P*] where *P* = (*F*_o_^2^ + 2*F*_c_^2^)/3
2817 reflections	(Δ/σ)_max_ < 0.001
232 parameters	Δρ_max_ = 0.18 e Å^−^^3^
2 restraints	Δρ_min_ = −0.24 e Å^−^^3^

### Special details

**Table:**

Geometry. All e.s.d.'s (except the e.s.d. in the dihedral angle between two l.s. planes) are estimated using the full covariance matrix. The cell e.s.d.'s are taken into account individually in the estimation of e.s.d.'s in distances, angles and torsion angles; correlations between e.s.d.'s in cell parameters are only used when they are defined by crystal symmetry. An approximate (isotropic) treatment of cell e.s.d.'s is used for estimating e.s.d.'s involving l.s. planes.
Refinement. Refinement of *F*^2^ against ALL reflections. The weighted *R*-factor *wR* and goodness of fit *S* are based on *F*^2^, conventional *R*-factors *R* are based on *F*, with *F* set to zero for negative *F*^2^. The threshold expression of *F*^2^ > σ(*F*^2^) is used only for calculating *R*-factors(gt) *etc*. and is not relevant to the choice of reflections for refinement. *R*-factors based on *F*^2^ are statistically about twice as large as those based on *F*, and *R*- factors based on ALL data will be even larger.

### Fractional atomic coordinates and isotropic or equivalent isotropic displacement parameters (Å^2^)

**Table:**

	*x*	*y*	*z*	*U*_iso_*/*U*_eq_	
N1	0.2035 (2)	0.17748 (14)	0.5732 (2)	0.0418 (6)	
N2	0.2552 (2)	0.24273 (14)	0.4891 (2)	0.0411 (6)	
N3	0.4644 (2)	0.60204 (17)	0.2185 (3)	0.0537 (7)	
O1	0.06218 (18)	0.12126 (14)	0.7791 (2)	0.0601 (6)	
O2	0.25885 (18)	0.33099 (12)	0.6893 (2)	0.0533 (6)	
O3	0.5244 (2)	0.58734 (14)	0.1093 (3)	0.0714 (7)	
O4	0.4424 (2)	0.67498 (14)	0.2634 (3)	0.0884 (9)	
C1	0.1483 (2)	0.02893 (16)	0.5926 (3)	0.0360 (7)	
C2	0.0776 (2)	0.04214 (18)	0.7164 (3)	0.0438 (7)	
C3	0.0168 (3)	−0.0276 (2)	0.7829 (3)	0.0561 (9)	
H3	−0.0314	−0.0177	0.8648	0.067*	
C4	0.0283 (3)	−0.1091 (2)	0.7281 (4)	0.0575 (9)	
H4A	−0.0136	−0.1540	0.7724	0.069*	
C5	0.1020 (2)	−0.12764 (18)	0.6053 (3)	0.0462 (8)	
C6	0.1163 (3)	−0.21292 (19)	0.5488 (4)	0.0634 (10)	
H6	0.0747	−0.2584	0.5920	0.076*	
C7	0.1890 (3)	−0.2291 (2)	0.4333 (4)	0.0697 (10)	
H7	0.1977	−0.2854	0.3981	0.084*	
C8	0.2511 (3)	−0.1615 (2)	0.3673 (4)	0.0650 (10)	
H8	0.3013	−0.1730	0.2879	0.078*	
C9	0.2395 (3)	−0.07840 (17)	0.4173 (3)	0.0504 (8)	
H9	0.2825	−0.0344	0.3718	0.060*	
C10	0.1632 (2)	−0.05776 (16)	0.5373 (3)	0.0377 (7)	
C11	0.2028 (2)	0.10178 (16)	0.5167 (3)	0.0377 (7)	
H11	0.2380	0.0929	0.4248	0.045*	
C12	0.2780 (2)	0.31916 (16)	0.5567 (3)	0.0377 (7)	
C13	0.3284 (2)	0.38988 (16)	0.4623 (3)	0.0341 (6)	
C14	0.3080 (2)	0.47496 (16)	0.5080 (3)	0.0416 (7)	
H14	0.2641	0.4849	0.5935	0.050*	
C15	0.3512 (2)	0.54420 (17)	0.4296 (3)	0.0436 (8)	
H15	0.3365	0.6007	0.4604	0.052*	
C16	0.4170 (2)	0.52799 (16)	0.3039 (3)	0.0386 (7)	
C17	0.4401 (2)	0.44479 (17)	0.2556 (3)	0.0431 (7)	
H17	0.4845	0.4355	0.1702	0.052*	
C18	0.3963 (2)	0.37526 (17)	0.3363 (3)	0.0418 (7)	
H18	0.4124	0.3189	0.3060	0.050*	
H2	0.263 (3)	0.2326 (19)	0.3912 (13)	0.080*	
H1	0.100 (3)	0.1599 (15)	0.730 (3)	0.080*	

### Atomic displacement parameters (Å^2^)

**Table:**

	*U*^11^	*U*^22^	*U*^33^	*U*^12^	*U*^13^	*U*^23^
N1	0.0548 (16)	0.0325 (13)	0.0381 (15)	−0.0026 (11)	0.0055 (12)	0.0056 (12)
N2	0.0596 (16)	0.0313 (13)	0.0326 (14)	−0.0049 (11)	0.0078 (13)	0.0009 (12)
N3	0.0514 (17)	0.0484 (18)	0.061 (2)	−0.0082 (13)	0.0051 (15)	0.0071 (15)
O1	0.0646 (16)	0.0614 (15)	0.0547 (16)	0.0077 (12)	0.0191 (12)	−0.0034 (12)
O2	0.0846 (16)	0.0478 (12)	0.0279 (12)	−0.0042 (10)	0.0145 (11)	−0.0027 (9)
O3	0.0816 (18)	0.0724 (16)	0.0608 (16)	−0.0184 (13)	0.0259 (14)	0.0057 (13)
O4	0.106 (2)	0.0380 (13)	0.122 (2)	0.0020 (13)	0.0465 (17)	0.0116 (14)
C1	0.0366 (16)	0.0398 (17)	0.0317 (17)	0.0017 (13)	0.0015 (14)	0.0060 (13)
C2	0.0439 (18)	0.0465 (18)	0.0409 (19)	0.0049 (14)	0.0008 (16)	0.0026 (15)
C3	0.047 (2)	0.079 (2)	0.043 (2)	−0.0017 (17)	0.0121 (16)	0.0169 (18)
C4	0.053 (2)	0.052 (2)	0.067 (2)	−0.0131 (16)	−0.0026 (19)	0.0260 (18)
C5	0.0410 (18)	0.0447 (19)	0.053 (2)	−0.0038 (14)	−0.0023 (16)	0.0124 (16)
C6	0.068 (2)	0.037 (2)	0.085 (3)	−0.0094 (16)	−0.012 (2)	0.0134 (19)
C7	0.081 (3)	0.038 (2)	0.091 (3)	0.0013 (18)	−0.010 (2)	−0.006 (2)
C8	0.075 (2)	0.048 (2)	0.072 (3)	0.0029 (18)	0.005 (2)	−0.0115 (18)
C9	0.057 (2)	0.0370 (18)	0.058 (2)	−0.0003 (14)	0.0067 (17)	0.0002 (15)
C10	0.0387 (17)	0.0341 (16)	0.0402 (18)	−0.0008 (13)	−0.0045 (14)	0.0076 (14)
C11	0.0428 (17)	0.0367 (17)	0.0338 (17)	0.0012 (13)	0.0065 (13)	0.0058 (14)
C12	0.0427 (17)	0.0377 (17)	0.0329 (18)	0.0023 (13)	0.0033 (14)	−0.0011 (14)
C13	0.0376 (16)	0.0339 (16)	0.0307 (17)	−0.0016 (13)	0.0018 (13)	−0.0022 (13)
C14	0.0487 (18)	0.0394 (17)	0.0370 (18)	0.0017 (13)	0.0126 (14)	−0.0052 (14)
C15	0.0482 (18)	0.0344 (16)	0.048 (2)	0.0036 (13)	0.0071 (16)	−0.0034 (14)
C16	0.0380 (16)	0.0349 (16)	0.0428 (18)	−0.0053 (13)	0.0020 (14)	0.0011 (14)
C17	0.0462 (18)	0.0490 (18)	0.0344 (18)	−0.0047 (14)	0.0109 (14)	−0.0030 (15)
C18	0.0478 (18)	0.0369 (16)	0.0408 (19)	−0.0023 (13)	0.0053 (15)	−0.0082 (14)

### Geometric parameters (Å, °)

**Table:**

N1—C11	1.274 (3)	C6—C7	1.351 (4)
N1—N2	1.390 (3)	C6—H6	0.9300
N2—C12	1.350 (3)	C7—C8	1.392 (4)
N2—H2	0.898 (10)	C7—H7	0.9300
N3—O3	1.218 (3)	C8—C9	1.365 (4)
N3—O4	1.222 (3)	C8—H8	0.9300
N3—C16	1.479 (3)	C9—C10	1.420 (4)
O1—C2	1.357 (3)	C9—H9	0.9300
O1—H1	0.859 (10)	C11—H11	0.9300
O2—C12	1.227 (3)	C12—C13	1.496 (3)
C1—C2	1.388 (4)	C13—C18	1.391 (3)
C1—C10	1.437 (3)	C13—C14	1.395 (3)
C1—C11	1.453 (3)	C14—C15	1.371 (3)
C2—C3	1.411 (4)	C14—H14	0.9300
C3—C4	1.357 (4)	C15—C16	1.379 (4)
C3—H3	0.9300	C15—H15	0.9300
C4—C5	1.415 (4)	C16—C17	1.381 (3)
C4—H4A	0.9300	C17—C18	1.388 (3)
C5—C10	1.420 (3)	C17—H17	0.9300
C5—C6	1.420 (4)	C18—H18	0.9300
			
C11—N1—N2	116.7 (2)	C7—C8—H8	119.5
C12—N2—N1	117.8 (2)	C8—C9—C10	121.4 (3)
C12—N2—H2	125 (2)	C8—C9—H9	119.3
N1—N2—H2	117 (2)	C10—C9—H9	119.3
O3—N3—O4	123.6 (3)	C5—C10—C9	117.0 (2)
O3—N3—C16	118.7 (3)	C5—C10—C1	120.0 (3)
O4—N3—C16	117.7 (3)	C9—C10—C1	123.0 (2)
C2—O1—H1	110 (2)	N1—C11—C1	121.6 (3)
C2—C1—C10	118.9 (2)	N1—C11—H11	119.2
C2—C1—C11	120.6 (2)	C1—C11—H11	119.2
C10—C1—C11	120.5 (2)	O2—C12—N2	122.2 (2)
O1—C2—C1	122.8 (3)	O2—C12—C13	120.9 (2)
O1—C2—C3	116.5 (3)	N2—C12—C13	117.0 (2)
C1—C2—C3	120.7 (3)	C18—C13—C14	119.0 (2)
C4—C3—C2	120.3 (3)	C18—C13—C12	123.8 (2)
C4—C3—H3	119.9	C14—C13—C12	117.1 (2)
C2—C3—H3	119.9	C15—C14—C13	121.5 (3)
C3—C4—C5	122.0 (3)	C15—C14—H14	119.3
C3—C4—H4A	119.0	C13—C14—H14	119.3
C5—C4—H4A	119.0	C14—C15—C16	118.3 (2)
C4—C5—C10	118.1 (3)	C14—C15—H15	120.8
C4—C5—C6	122.4 (3)	C16—C15—H15	120.8
C10—C5—C6	119.5 (3)	C15—C16—C17	122.0 (2)
C7—C6—C5	121.2 (3)	C15—C16—N3	118.9 (3)
C7—C6—H6	119.4	C17—C16—N3	119.0 (3)
C5—C6—H6	119.4	C16—C17—C18	119.1 (3)
C6—C7—C8	119.9 (3)	C16—C17—H17	120.5
C6—C7—H7	120.1	C18—C17—H17	120.5
C8—C7—H7	120.1	C17—C18—C13	120.0 (2)
C9—C8—C7	120.9 (3)	C17—C18—H18	120.0
C9—C8—H8	119.5	C13—C18—H18	120.0

### Hydrogen-bond geometry (Å, °)

**Table:**

*D*—H···*A*	*D*—H	H···*A*	*D*···*A*	*D*—H···*A*
O1—H1···N1	0.86 (1)	1.85 (2)	2.599 (3)	144 (3)
N2—H2···O2^i^	0.90 (1)	2.06 (1)	2.923 (3)	160 (3)

Symmetry codes: (i) *x*, −*y*+1/2, *z*−1/2.
